# The Price of Precision: A Critical Review of Molecular Diagnostics in Glioma, From Guidelines to Global Disparities

**DOI:** 10.1002/acn3.70503

**Published:** 2026-07-28

**Authors:** Maria Guarnaccia, Sebastiano Cavallaro

**Affiliations:** ^1^ Institute for Biomedical Research and Innovation (IRIB) National Research Council (CNR) Catania Italy

**Keywords:** gliomas, global disparities, health economics, integrated diagnosis, precision medicine, tiered diagnostics, WHO Molecular Taxonomy

## Abstract

Gliomas have undergone a profound redefinition over the past decade, transitioning from morphology‐based entities to biologically coherent diseases defined by molecular alterations. The 2021 WHO Classification of Tumors of the Central Nervous System and its 2022 update formalize this shift, establishing integrated diagnosis as the global standard and identifying essential biomarkers—including *IDH* mutations, 1p/19q codeletion, *ATRX* loss, *TP53* mutation, *TERT* promoter mutation, *EGFR* amplification, and +7/−10 cytogenetics—that shape prognosis, therapeutic decision‐making, and clinical trial eligibility. In this review, we synthesize the current molecular taxonomy of gliomas and compare recommendations from major international guidelines (WHO, NCCN, EANO) and the Italian Association of Neuro‐Oncology (AINO). Recognizing that contemporary practice employs a complementary, purpose‐driven array of diagnostic modalities—from immunohistochemistry and PCR‐based methods to next‐generation sequencing and DNA methylation profiling—we critically examine the profound global disparities that hinder implementation, focusing on economic constraints, infrastructural limitations, regulatory barriers, and workforce shortages. By integrating health‐economic evidence, we demonstrate that the societal and financial burden of non‐implementation far exceeds the investment required for molecular testing. We propose a tiered implementation framework in which clinically actionable information is obtainable for most glioma patients through accessible, cost‐effective technologies, with more resource‐intensive modalities reserved for specific clinical scenarios. Ensuring equitable access to integrated diagnostics, defined as the right test for the right clinical question, is essential not only for precision neuro‐oncology but also for global healthcare justice and scientific progress.

**Abbreviations**
ADAPTR: Adapting Diagnostic Approaches for Practical Taxonomy in Resource‐Restrained Regions
AINO: Italian Association of Neuro‐Oncology
AOSNP: Asian Oceanian Society of Neuropathology
CBCA: Chinese Brain Cancer Association
CGCG: Chinese Glioma Cooperative Group
CME: Continuing Medical Education
CNS: central nervous system
dPCR: digital polymerase chain reaction
EANO: European Association of Neuro‐Oncology
FISH: fluorescence in situ hybridization
IHC: immunohistochemistry
LMICs: low‐ and middle‐income countries
MLPA: multiplex ligation‐dependent probe amplification
NCCN: National Comprehensive Cancer Network
NGS: next‐generation sequencing
PCV: procarbazine, lomustine, vincristine
qPCR: quantitative polymerase chain reaction
WHO: World Health Organization
WHO CNS5: WHO Classification of Tumors of the Central Nervous System, 5th Edition

## 1 Introduction: The Paradigm Shift in Glioma Classification

Gliomas are the most common and aggressive primary tumors of the central nervous system (CNS), representing a biologically heterogeneous group of neoplasms with substantial clinical and societal impact [1, 2]. Despite advances in neurosurgery, radiotherapy, and systemic therapies, outcomes remain poor, particularly for high‐grade gliomas, which are associated with rapid neurological decline and significant economic burden [1, 3].

For decades, glioma classification relied primarily on histopathology, a framework limited by inter‐observer variability and its inability to capture the biological diversity underlying clinical behavior [4, 5, 6]. The discovery of key molecular alterations—most notably *IDH* mutations and 1p/19q codeletion—has fundamentally reshaped the conceptual landscape of glioma taxonomy. Molecular profiling is now the primary determinant of diagnostic identity, culminating in the integrated diagnostic model formalized in the 2021 WHO Classification of Tumors of the Central Nervous System, fifth edition (WHO CNS5) [7, 8, 9].

This paradigm shift has improved diagnostic precision, prognostication, and therapeutic stratification. However, despite broad scientific consensus, real world implementation remains fragmented [10]. Access to molecular diagnostics varies dramatically across countries, healthcare systems, and even within nations, creating a widening gap between diagnostic capability and clinical need.

Importantly, contemporary neuro‐oncological practice employs a complementary array of diagnostic technologies, selected according to the specific diagnostic question, available resources, and local expertise. Immunohistochemistry (IHC), fluorescence in situ hybridization (FISH), PCR‐based methods including MLPA and digital PCR, targeted next‐generation sequencing panels, and genome‐wide DNA methylation profiling each occupy distinct and often non‐overlapping roles within an integrated diagnostic workflow [11, 12, 13]. This review does not advocate a linear progression from “basic” to “advanced” technologies, but rather a purpose‐driven, tiered approach in which the clinical question determines the most appropriate modality. Presenting NGS or methylation arrays as universal gold standards reflects the practice of a limited number of well‐resourced centers and does not align with global diagnostic realities, as documented by recent surveys from the Asian Oceanian Society of Neuropathology (AOSNP) and the Lancet Commission on Diagnostics [14].

This review synthesizes the current molecular taxonomy of gliomas, compares major international guidelines, and critically examines the global disparities that hinder implementation. By integrating health‐economic evidence and real‐world data, we highlight the urgent need for sustainable, equitable diagnostic strategies that match technological approaches to clinical need.

## 2 The WHO Molecular Taxonomy: From the 2016 to the 2021/2022 Editions

The WHO CNS5 and its 2022 update for adult‐type diffuse gliomas formalize a “layered” diagnostic report, prioritizing molecularly defined types over standalone histology [15, 16]. This framework integrates histological and molecular information into a single, clinically actionable diagnosis.

### 2.1 Adult‐Type Diffuse Gliomas

This category is now primarily defined by three molecularly distinct entities [17].

*Astrocytoma*, *IDH‐mutant* is characterized by *IDH1/2* mutations. These tumors typically show concurrent mutations in *ATRX* and *TP53* and absence of 1p/19q codeletion. They are graded (CNS WHO grades 2–4) based on histological features (mitotic activity, microvascular proliferation, necrosis) and/or the presence of *CDKN2A/B* homozygous deletion, which confers a grade 4 designation regardless of histology [18, 19].

*Oligodendroglioma*, *IDH‐mutant and 1p/19q‐codeleted* is defined by the canonical dual signature of *IDH* mutation and whole‐arm 1p/19q codeletion. Additional alterations include *TERT* promoter mutations, *CIC* and *FUBP1* mutations, and *NOTCH1* pathway involvement. Tumors are classified as grade 2 or 3 based on histology [18, 20].

*Glioblastoma*, *IDH‐wildtype* represents the most common and aggressive adult glioma. Diagnosis can be established in an *IDH*‐wildtype diffuse glioma even without classic histological features if one of the following is present: *TERT* promoter mutation, *EGFR* amplification, or +7/−10 cytogenetic signature. *MGMT* promoter methylation remains a key predictive biomarker for temozolomide response [18, 20].

### 2.2 Pediatric‐Type Diffuse Gliomas

Pediatric‐type gliomas represent biologically distinct entities driven by alterations in histone H3 genes or MAPK/ERK pathway dysregulation. Key subtypes include: Diffuse midline glioma, H3 K27‐altered [21], Diffuse hemispheric glioma, H3 G34‐mutant [22], Diffuse pediatric‐type high‐grade glioma, H3‐wildtype and *IDH*‐wildtype [23], and Pediatric low‐grade gliomas driven by *MYB/MYBL1*, *BRAF*, or *FGFR1* alterations [24]. These entities highlight the importance of molecular profiling in defining biologically coherent disease categories and underscore the need for diagnostic approaches tailored to the specific alteration being sought.

## 3 International and National Guidelines: A Comparative Analysis

The WHO CNS5, commonly known as the “Blue Book”, provides the definitive diagnostic criteria and standardized nomenclature, mandating the integration of molecular information and setting the global standard for clinical care and research [20].

### 3.1 International and National Guideline Recommendations

The primary goal of these guidelines is to translate scientific advances into reliable clinical practice, improving outcomes for patients with glioma [25, 26]. Table 1 summarizes the essential molecular markers recommended by major international and national guidelines for adult‐type diffuse gliomas.

**TABLE 1:** Molecular marker recommendations in major guidelines for adult diffuse gliomas.

Molecular marker	Primary role	WHO CNS5 (2021)	NCCN guidelines	EANO guidelines	AINO guidelines (Italy)
IDH1/2 mutation	Diagnostic (type)	Essential	Essential	Essential	Essential (Core test)
1p/19q codeletion	Diagnostic (type)	Essential (if *IDH*‐mut)	Essential (if *IDH*‐mut)	Essential (if *IDH*‐mut)	Essential (Core test)
ATRX loss/mutation	Diagnostic (supportive)	Recommended	Recommended	Recommended	Recommended (IHC/NGS)
TP53 mutation	Diagnostic (supportive)	Recommended	Recommended	Recommended	Recommended (IHC/NGS)
TERT promoter mut	Diagnostic & Prognostic	Required for GBM^a^	Recommended	Recommended	Recommended
MGMT methylation	Predictive (therapy)	Recommended for GBM	Recommended for GBM	Recommended for GBM	Recommended for GBM
CDKN2A/B del	Grading (Astro, *IDH*‐mut)	Required for Grade 4	Recommended	Recommended	Recommended
EGFR amplification	Diagnostic (*IDH*‐wt GBM)	Required for GBM^a^	Recommended	Recommended	Recommended
+7/−10	Diagnostic (*IDH*‐wt GBM)	Required for GBM^a^	Recommended	Recommended	Recommended
H3 K27M	Diagnostic (type)	Essential in midline	Essential in midline	Essential in midline	Essential in midline

Abbreviations: AINO, Italian Association of Neuro‐Oncology; Astro, astrocytoma; EANO, European Association of Neuro‐Oncology; GBM, glioblastoma; NCCN, National Comprehensive Cancer Network.

^a^ Presence of one of these molecular markers, along with *IDH*‐wildtype status, suffices for a diagnosis of Glioblastoma, *IDH*‐wildtype.

Although imaging interpretation is not formally incorporated into the WHO CNS5 classification, it remains critically important in the diagnostic pathway, particularly for surgical planning, assessment of tumor extent, and evaluation of treatment response or progression [16].

### 3.2 Italian Guidelines: The AINO Recommendations

The AINO has published specific national guidelines that closely mirror and operationalize the WHO and EANO frameworks [27]. Key features include:

- *Mandatory core tests*: AINO defines *IDH1/2* sequencing and 1p/19q status as essential to distinguish *IDH*‐mutant from *IDH*‐wildtype gliomas, and to identify the oligodendroglioma subtype.
- *Integrated grading*: the guidelines explicitly adopt the WHO grading system, incorporating *CDKN2A/*B homozygous deletion to designate *IDH*‐mutant astrocytoma as grade 4 and *MGMT* promoter methylation to guide treatment decisions.
- *Complementary diagnostic approach*: AINO recognizes the value of immunohistochemistry and FISH as first‐line tools, while also recommending consideration of targeted NGS panels and DNA methylation profiling for diagnostically challenging cases. The guidelines emphasize that the choice of technique should be guided by the specific clinical question and local resources.
- *Operational pathways*: AINO provides practical flowcharts for pathologists and clinicians, promoting standardized reporting across Italian centers. “Operational pathways” here refer to the structured sequence of diagnostic steps, from tissue handling through molecular testing to integrated reporting that ensures consistent, guideline‐concordant care.

## 4 Economic and Societal Costs: The Price of Implementation Versus the Burden of Inaction

The economic implications of implementing integrated molecular diagnostics extend far beyond the price of individual tests. A comprehensive health‐economic perspective must consider the entire diagnostic–therapeutic continuum, including the downstream consequences of misclassification, ineffective treatments, and accelerated disease progression [28].

### 4.1 Direct Costs and Investments: A Tiered Perspective

A fundamental principle of sustainable diagnostics is that the choice of technology should match the clinical question. Table 2 presents representative cost estimates for commonly used diagnostic modalities, though actual costs vary substantially among healthcare systems, reimbursement policies, laboratory workflows, and testing volumes. Recent analyses estimate average costs of approximately €290 for IHC, €795 for FISH, €261 for PCR/qPCR, and €1538 for targeted NGS panels [29, 30].

**TABLE 2:** Representative costs of commonly used molecular diagnostic techniques.

Technique	Estimated cost per test (EUR)*	Complexity
Immunohistochemistry (IHC)	~290	Low
Fluorescence In Situ Hybridization (FISH)	~795	Medium
PCR/qPCR	~261	Low–Medium
MLPA	~300–500	Medium
Digital PCR (dPCR)	~350–600	Medium
Targeted NGS Panel	~1538	High
DNA Methylation Profiling	~2000–3000	Very High

* Costs are approximate and vary substantially among healthcare systems, reimbursement policies, and testing volumes [4, 5].

In many healthcare systems, a tiered diagnostic strategy—based on IHC, MLPA, qPCR, or digital PCR (dPCR)—provides substantial clinical value at a fraction of the cost of comprehensive genomic profiling. These methods are particularly well‐suited for essential biomarkers such as 1p/19q codeletion, *CDKN2A/B* deletion, *TERT* promoter mutation, *EGFR* amplification, and +7/−10 cytogenetics [11]. Importantly, for the majority of glioma patients, these Tier 1 approaches yield all clinically actionable information needed for diagnosis, grading, and treatment planning. Targeted NGS panels and genome‐wide methylation profiling, while invaluable for diagnostically challenging cases and research settings, are best understood as Tier 2 and Tier 3 resources to be deployed when first‐line methods are insufficient to resolve the clinical question [31, 32]. This tiered conceptualization does not represent a compromise for resource‐limited settings; rather, it reflects a clinically rational, purpose‐driven approach applicable across all healthcare contexts.

### 4.2 The High Cost of Non‐Implementation: Patient and Societal Burden

The financial burden of failing to implement molecular diagnostics is significantly greater than the cost of testing (Table 3) [33]. Misclassification leads to inappropriate therapies, unnecessary toxicity, and accelerated disease progression [34, 35]. For example, a single cycle of temozolomide in an *MGMT*‐unmethylated glioblastoma patient, who derives minimal benefit, often exceeds the cost of a full targeted NGS panel.

**TABLE 3:** Comparative cost analysis: Molecular testing vs. consequences of its absence.

Cost Category	With Integrated Molecular Diagnosis	Without Integrated Molecular Diagnosis (Histology‐Only)
Upfront diagnostic cost	Variable: ~€300 for IHC/PCR to ~€1500 for NGS panel	Low: ~€200–500 for basic histology + limited IHC
Therapy cost (First Line)	Precise: Therapy matched to biology (e.g., PCV for 1p/19q‐codeleted oligodendroglioma)	Empirical: “One‐size‐fits‐all” approach (e.g., temozolomide for all high‐grade gliomas)
Cost of ineffective therapy	Low: Reduced likelihood of administering useless, toxic treatment	Very High: Paying for expensive drugs/radiotherapy that offer no benefit, plus managing side effects
Cost of disease progression	Potentially Delayed/Reduced: More effective initial control may delay costly late‐stage care	High: Faster progression leads to earlier need for salvage therapy, hospitalization, and palliative care
Societal cost (Productivity)	Potentially Higher: Patients may maintain functional status longer	Very High: Rapid functional decline leads to loss of employment, disability support, and caregiver burden

Non‐implementation also increases emergency admissions, early initiation of salvage therapies, and long‐term disability costs [34, 35, 36]. At the societal level, premature functional decline results in loss of productivity, caregiver burden, and increased reliance on social support systems [37]. These cumulative costs far outweigh the initial investment required for integrated diagnostics, regardless of the specific technological modality employed.

## 5 The Downstream Consequences: How Missing Markers Mismanages Therapy and Stifles Innovation

### 5.1 Limits in Routine Therapy Application

The absence of molecular diagnostics directly compromises therapeutic decision‐making at multiple levels:

- *MGMT promoter methylation*: without this test, temozolomide is administered empirically to all glioblastoma patients, despite limited benefit in approximately 60% of unmethylated cases [38, 39]. The cost of a single futile cycle of temozolomide frequently exceeds that of the *MGMT* methylation assay itself.
- *1p/19q codeletion*: the standard‐of‐care for oligodendroglioma is procarbazine, lomustine, and vincristine (PCV) chemotherapy plus radiation, which prolongs survival compared to radiation alone [40, 41]. Without testing, patients may receive suboptimal treatment, with consequences measured in years of life lost.
- *IDH mutation*: *IDH*‐mutant gliomas have distinct prognostic and therapeutic implications, including eligibility for IDH inhibitors [42, 43]. Lack of testing denies patients access to targeted therapies and molecularly stratified clinical trials.

### 5.2 Stifling Innovation: The Impact on Research and Clinical Trial Access

The lack of molecular diagnostics represents a critical bottleneck for therapeutic research and patient access to innovation [44]. Contemporary clinical trials are increasingly molecularly driven: centers without testing capabilities cannot screen or enroll eligible patients, creating “diagnostic deserts” and “trial deserts” – geographic regions where patients are systematically excluded from cutting‐edge research due to lack of diagnostic infrastructure rather than clinical ineligibility [45, 46]. Historical trials conducted without molecular stratification are now difficult to interpret, as treatment benefits are often confined to molecular subgroups [47, 48]. Similarly, biobanks without molecular annotation are of limited value for discovering new biomarkers [49]. On a global scale, this leads to a skewed understanding of the disease: the published molecular landscape of gliomas is heavily biased toward populations served by well‐resourced centers, potentially overlooking features unique to underrepresented regions [50].

## 6 Global Implementation: A Landscape of Heterogeneity and Disparity

Implementation of integrated molecular diagnostics varies widely across regions, reflecting differences in infrastructure, reimbursement, workforce expertise, and national policies [51]. It is important to recognize that the diagnostic workflows described in the Western literature—often centered on NGS panels and methylation arrays—reflect the practice of a minority of well‐resourced centers globally. Recent surveys indicate that these technologies remain inaccessible in most countries and are frequently restricted to major urban tertiary centers even within high‐income nations [14].

### 6.1 High‐Income Countries

In Europe, most tertiary centers adopt a tiered, complementary diagnostic approach, combining histopathology, IHC, PCR‐based assays, MLPA, and FISH as first‐line modalities. Targeted NGS panels are used selectively for cases requiring broader molecular characterization, and genome‐wide DNA methylation profiling is available primarily in specialized referral centers [11, 52]. Significant variation exists among and within European nations, with rural and community hospitals often having limited access to NGS and methylation platforms.

In the United States, integrated molecular diagnosis is widely implemented in academic centers, supported by broad access to targeted NGS panels, methylation profiling, and molecularly stratified clinical trials [53, 54]. However, substantial disparities persist between academic institutions and community hospitals, particularly regarding bioinformatics infrastructure, reimbursement mechanisms, and access to specialized neuropathology expertise [55, 56].

### 6.2 Asia

In China, the clinical practice guidelines for adult diffuse gliomas have been revised by the Chinese Glioma Cooperative Group (CGCG), the Society for Neuro‐Oncology of China (SNO‐China), and the Chinese Brain Cancer Association (CBCA) [57]. Genomic oncology capacity has expanded rapidly, with widespread adoption of large commercial NGS panels in major urban centers [13]. However, access remains heterogeneous, with significant disparities between metropolitan and rural regions [54]. The ratio of NGS‐capable centers to population varies by an order of magnitude between coastal metropolitan areas and inland provinces.

Japan has progressively integrated molecular diagnostics through coordinated national cancer networks, emphasizing standardized workflows and high‐quality neuropathology services. The OncoGuide NCC Oncopanel and other targeted assays are reimbursed through the national health insurance system, facilitating broader access [53].

South Korea has incorporated targeted NGS panels into tertiary centers, with national reimbursement for approved panels since 2017. However, access remains uneven across regions, with concentration in Seoul and other major cities [58].

### 6.3 Low‐ and Middle‐Income Countries (LMICs)

According to the AOSNP‐ADAPTR survey, fewer than 15% of centers in LMICs have access to NGS, and methylation profiling is virtually absent. Most institutions rely on IHC and limited PCR‐based assays, perpetuating diagnostic inequities and limiting access to clinical trials [14]. This situation creates what we term “a two‐tiered neuro‐oncology”: a system in which access to accurate molecular diagnosis, targeted therapies, and clinical trials is determined not by clinical need but by geographic location and healthcare resources.

The choice of technique directly impacts cost, turnaround time, and diagnostic breadth (Table 4). These techniques should be understood as complementary rather than hierarchical: each has specific strengths and limitations, and their selection depends on the diagnostic question, available resources, and local expertise. For example, MLPA remains particularly useful compared to targeted NGS for assessing whole‐arm 1p/19q codeletion, while dPCR offers exceptional sensitivity for low‐allele‐fraction variants such as *TERT* promoter mutations [59]. No single modality addresses all diagnostic needs.

**TABLE 4:** Complementary molecular diagnostic techniques used in WHO CNS glioma classification: Principal applications, strengths, and limitations.

Technique	Typical applications	Turnaround time	Infrastructure requirements	Key advantages	Key limitations	Relative cost & complexity
Immunohistochemistry (IHC)	IDH1 R132H, ATRX, p53, H3 K27M, Ki‐67	1–2 days	Standard pathology laboratory; microscope; trained pathologist	Fast, inexpensive, integrates with histology; high sensitivity for specific epitopes	Limited to specific protein epitopes; semi‐quantitative; cannot detect non‐canonical mutations	Low cost, low complexity
Fluorescence In Situ Hybridization (FISH)	1p/19q deletion, *EGFR* amplification	2–5 days	Fluorescence microscope; dedicated probes; specialized personnel	Visual, cell‐specific, works on FFPE; high specificity	Probes limited to specific regions; may miss whole‐arm events; labor‐intensive interpretation	Medium cost, medium complexity
Pyrosequencing/Sanger	*IDH1/2*, *TERT* promoter, *H3*, *BRAF* V600E	2–5 days	Molecular diagnostics laboratory; sequencing equipment	Quantitative, accurate for known hotspots; high sensitivity and specificity	Limited multiplexing capability; requires prior knowledge of target	Medium cost, medium complexity
MLPA/qPCR	1p/19q whole‐arm assessment, chromosome +7/−10 assessment, *EGFR* amplification, *CDKN2A/B* deletion, *TERT* promoter mutations, *IDH1/2* status, *MGMT* promoter methylation	1–3 days	Basic molecular laboratory; PCR instrumentation	Excellent for whole‐arm losses; high sensitivity; cost‐effective; minimal bioinformatics	Limited to pre‐designed targets; requires careful normalization	Low–Medium cost, medium complexity
Digital PCR (dPCR)	*IDH1/2*, *TERT* promoter, *EGFR* variants, *CDKN2A* homozygous deletion, *H3‐3A*, *MGMT* methylation	1–3 days	Specialized dPCR platform; trained personnel	Ultra‐sensitive; excellent for low allele frequency variants; highly reproducible; minimal bioinformatics requirements	Limited multiplexing; requires prior knowledge of target; platform cost is moderate	Medium cost, medium complexity
Next‐Generation Sequencing (NGS) Panel	Simultaneous analysis of multiple genes; detection of mutations, copy number changes, fusions	5–20 days	Sequencing platform; bioinformatics infrastructure; specialized staff	Comprehensive, high‐throughput, efficient use of tissue; can detect unexpected variants	High upfront cost; requires bioinformatics expertise; longer turnaround time; may miss whole‐arm events	High cost, high complexity
DNA Methylation Profiling	Genome‐wide methylation pattern; diagnostic classification	7–21 days	Advanced genomic laboratory; computational analysis pipeline	Powerful for rare/ambiguous tumors; high diagnostic accuracy; gold standard for challenging cases	Extremely high cost; limited availability; requires specialized bioinformatics; long turnaround time	Very high cost, very high complexity

## 7 Barriers to Implementation: a Systematic Analysis

The obstacles to universal implementation are multifactorial and interlinked, forming a self‐reinforcing cycle that hinders progress (Table 5) [45]. High costs and lack of funding (Economic) lead to reliance on a limited range of techniques and a shortage of specialized personnel (Human Resources). This, in turn, results in non‐standardized processes (Pre‐analytical) and an inability to meet regulatory standards for new test adoption (Regulatory). The lack of exposure to the full spectrum of diagnostic modalities then limits the development of local expertise and knowledge, perpetuating the cycle.

**TABLE 5:** Key barriers to the implementation of integrated molecular diagnosis.

Category of barrier	Specific challenges	Consequences
Economic & Infrastructural	High cost of advanced platforms and reagents; lack of stable funding/reimbursement; unreliable supply chains for consumables	Restricted test menu; disparity between centers; dependence on send‐out testing with long turnaround times
Human Resources & Expertise	Shortage of neuropathologists, molecular biologists, bioinformaticians; lack of continuous medical education (CME); brain drain to high‐income countries	Incorrect test selection, technical errors, misinterpretation of results; inability to operationalize guidelines
Pre‐analytical & Technical	Non‐standardized tissue fixation and procurement; lack of quality control programs; inadequate sample tracking	Sample failure for molecular tests; inaccurate results; inability to repeat or confirm findings
Regulatory & Systemic	Bureaucratic delays in approving/reimbursing new tests; fragmented healthcare systems; absence of national diagnostic strategies	Long lag time for technology adoption; inconsistent patient access; duplication of scarce resources
Knowledge & Standardization	Variable interpretation of guidelines; lack of standardized reporting formats; limited participation in external quality assessment schemes	Inconsistent diagnostic criteria applied across centers; clinical confusion; inability to pool data for research

Furthermore, patients and caregivers face additional barriers from poor communication and fragmented care pathways, which can undermine shared decision‐making and timely access to appropriate treatment [60, 61].

## 8 A Call for Action: Toward Sustainable and Equitable Implementation

Achieving equitable implementation, as outlined by the WHO CNS5, means ensuring fair access to high‐quality healthcare for all [62, 63]. In the context of glioma diagnostics, equity does not require that every center possess NGS and methylation platforms. Rather, it requires that every patient have access to the right test for their specific clinical question, delivered in a timely manner and interpreted by qualified personnel. Addressing this requires a coordinated, multi‐level strategy.

### 8.1 A Tiered, Purpose‐Driven Implementation Framework

We propose a three‐tiered framework for glioma molecular diagnostics, designed to match technological approach to clinical need while maximizing equitable access:

- *Tier 1 (Essential, universally accessible)*: Histopathology with immunohistochemistry (IDH1 R132H, ATRX, p53, H3 K27M, Ki‐67), combined with qPCR, MLPA, or dPCR for 1p/19q codeletion, *CDKN2A/B* deletion, *TERT* promoter mutation, *EGFR* amplification, +7/−10 cytogenetics, and *MGMT* promoter methylation. This tier provides clinically actionable information for the large majority of glioma patients at relatively low cost, with minimal bioinformatics requirements and turnaround times of 1–5 days.
- *Tier 2 (Targeted, regionally available)*: Targeted NGS panels for cases requiring broader molecular characterization, such as *IDH*‐wildtype gliomas without canonical alterations, suspected rare fusions, or patients eligible for molecularly stratified clinical trials. These panels should be accessible through regional hub laboratories.
- *Tier 3 (Specialized, centralized)*: Genome‐wide DNA methylation profiling for diagnostically challenging tumors where Tier 1 and Tier 2 approaches yield ambiguous results. This modality is appropriately concentrated in reference centers serving large populations.

This framework is not a temporary compromise for resource‐limited settings. It represents a clinically rational, sustainable model in which the diagnostic question—not the available technology—determines the appropriate test. Even in well‐resourced centers, the majority of glioma diagnoses are established using Tier 1 methods, with Tiers 2 and 3 reserved for specific indications.

### 8.2 Practical Strategies for Implementation

Several practical strategies can facilitate the implementation of integrated molecular diagnostics across diverse settings:

- *Create shared resource networks*: Establishing regional molecular diagnostic hubs—“hub‐and‐spoke” networks as advocated by the Lancet Commission on Diagnostics—maximizes the use of equipment and expertise while reducing per‐test costs through economies of scale [32, 64]. Telepathology and digital neuropathology platforms allow remote interpretation of histological and molecular data by specialized centers, overcoming shortages of trained neuropathologists in resource‐limited regions [65].
- *Integrate diagnostics into health economics*: advocate that governments and payers use robust data (Table 3) demonstrating that diagnostic investment reduces long‐term costs by avoiding ineffective therapies, preventing costly disease progression, and preserving societal productivity [33, 66]. Health technology assessment frameworks should explicitly consider the cost of non‐implementation.
- *Mandate diverse representation in research*: supporting the export of samples to centralized laboratories and establishing mechanisms for sample referral can ensure patients in all regions have opportunities to participate in global research, reducing the current bias in the published molecular landscape of gliomas [67].
- *Invest in workforce development*: target training for pathologists, neurosurgeons, oncologists, and radiologists in the interpretation and clinical application of molecular diagnostics. The MNP Outreach initiative, which aims to establish modern molecular cancer diagnostics in low‐income countries through training programs and centers of excellence, including free DNA methylation testing for over 300 patients annually in Pakistan, provides a model for such efforts [68].
- *Focus on predictive planning*: allocate resources effectively to streamline operations and enable health systems to adapt to evolving diagnostic needs [69]. National diagnostic strategies should anticipate technological evolution while prioritizing currently achievable standards.

### 8.3 Recommendations for WHO Action

The WHO is uniquely positioned to accelerate equitable implementation of integrated glioma diagnostics globally. We recommend that the WHO:

- Promote the establishment of regional reference laboratories, shared testing networks, and technology‐transfer partnerships to extend diagnostic capacity beyond well‐resourced centers [70].
- Develop and disseminate a minimum diagnostic standard for glioma, achievable with Tier 1 technologies, to ensure that no patient is managed without essential molecular information regardless of geographic location
- Support affordable diagnostic technologies through prequalification programs, negotiated pricing frameworks for reagents, and incentives for manufacturers to develop platforms suited to resource‐limited settings [71].
- Facilitate targeted training programs for pathologists, neurosurgeons, oncologists, and radiologists in LMICs, leveraging partnerships with international professional societies.

## 9 Conclusion

The molecular taxonomy of gliomas is a cornerstone of modern neuro‐oncology. Its implementation is not merely a technical upgrade but a fundamental issue of healthcare justice, economic efficiency, and scientific progress [55]. The upfront costs of diagnostics are dwarfed by the immense human and financial burdens of diagnostic inaccuracy.

A critical conceptual clarification is essential: the goal of equitable implementation is not universal access to NGS and methylation arrays. The goal is universal access to the right test for the clinical question—which, for the majority of glioma patients worldwide, can be achieved through cost‐effective, accessible Tier 1 technologies including IHC, qPCR, MLPA, and dPCR. Targeted NGS and methylation profiling are invaluable tools for specific clinical scenarios, and their availability should be expanded through regional hub‐and‐spoke networks, but their current inaccessibility in most of the world should not be used to justify diagnostic nihilism. An accurate, clinically actionable diagnosis is achievable today for most glioma patients using existing, affordable technologies, provided the political will and organizational frameworks exist to deliver them.

The alternative is the emergence and consolidation of a two‐tiered neuro‐oncology system in which access to accurate molecular diagnosis, targeted therapies, and clinical trials is determined not by clinical need but by geographic location and healthcare resources. Through strategic, tiered implementation, international collaboration, and vigorous advocacy, the global neuro‐oncology community must work toward a future in which every patient receives a diagnosis that not only names their disease but also accurately characterizes its biological nature.

## Author Contributions

Conceptualization: M.G. and S.C. Writing – original draft: M.G. and S.C.; Funding acquisition: S.C.; Writing – review and editing: S.C.

## Funding

This work was supported by the National Plan for Complementary Investments to the NRRP, project “D34H‐Digital Driven Diagnostics, prognostics and therapeutics for sustainable Health care” (project code: PNC0000001), Spoke 4, funded by the Italian Ministry of University and Research.

## Conflicts of Interest

The authors declare no conflicts of interest.

## Data Availability

The authors have nothing to report.
